# Identifying cortical structure markers of resilience to adversity in young people using surface-based morphometry

**DOI:** 10.1093/scan/nsae006

**Published:** 2024-01-27

**Authors:** Harriet Cornwell, Nicola Toschi, Catherine Hamilton-Giachritsis, Marlene Staginnus, Areti Smaragdi, Karen Gonzalez-Madruga, Nuria Mackes, Jack Rogers, Anne Martinelli, Gregor Kohls, Nora Maria Raschle, Kerstin Konrad, Christina Stadler, Christine M Freitag, Stephane A De Brito, Graeme Fairchild

**Affiliations:** Department of Psychology, University of Bath, 10 West, Claverton Down, Bath, Somerset BA2 7AY, UK; Department of Biomedicine and Prevention, University of Rome ‘Tor Vergata’, Facoltà di Medicina e Chirurgia, Viale Montpellier, Rome 1 – 00133, Italy; Martinos Center for Biomedical Imaging and Harvard Medical School, 149 13th Street Charlestown, Boston, MA 02129, USA; Department of Psychology, University of Bath, 10 West, Claverton Down, Bath, Somerset BA2 7AY, UK; Department of Psychology, University of Bath, 10 West, Claverton Down, Bath, Somerset BA2 7AY, UK; Child Development Institute, 197 Euclid Ave., Toronto, Ontario, M6J 2J8, Canada; Department of Psychology, Middlesex University, The Burroughs, Hendon, London NW4 4BT, UK; Department of Neuroimaging, Institute of Psychiatry, Psychology and Neuroscience, King’s College London, De Crespigny Park, London SE5 8AF, UK; Centre for Human Brain Health, School of Psychology, University of Birmingham, Edgbaston, Birmingham B15 2TT, UK; Department of Child and Adolescent Psychiatry, Psychosomatics and Psychotherapy, University Hospital Frankfurt, Goethe University, Deutschordenstrasse 50, Frankfurt am Main 60528, Germany; Fresenius University of Applied Sciences, School of Psychology, Marienburgstrasse 6, Frankfurt am Main 60528, Germany; Child Neuropsychology Section, Department of Child and Adolescent Psychiatry, Psychosomatics and Psychotherapy, University Hospital, RWTH Aachen, Pauwelsstrasse 30, Aachen 52074, Germany; Department of Child and Adolescent Psychiatry, Faculty of Medicine, TU Dresden, Fetscherstrasse 74, Dresden 01307, Germany; Department of Child and Adolescent Psychiatry, University of Basel, Psychiatric University Hospital, Wilhelm Klein-Strasse 27, Basel 4002, Switzerland; Jacobs Center for Productive Youth Development at the University of Zurich, Andreasstrasse 15, Zurich 8050, Switzerland; Child Neuropsychology Section, Department of Child and Adolescent Psychiatry, Psychosomatics and Psychotherapy, University Hospital, RWTH Aachen, Pauwelsstrasse 30, Aachen 52074, Germany; JARA-Brain Institute II, Molecular Neuroscience and Neuroimaging, RWTH Aachen and Research Centre Juelich, Wilhelm-Johnen-Straße, Juelich 52425, Germany; Department of Child and Adolescent Psychiatry, University of Basel, Psychiatric University Hospital, Wilhelm Klein-Strasse 27, Basel 4002, Switzerland; Department of Child and Adolescent Psychiatry, Psychosomatics and Psychotherapy, University Hospital Frankfurt, Goethe University, Deutschordenstrasse 50, Frankfurt am Main 60528, Germany; Centre for Human Brain Health, School of Psychology, University of Birmingham, Edgbaston, Birmingham B15 2TT, UK; Department of Psychology, University of Bath, 10 West, Claverton Down, Bath, Somerset BA2 7AY, UK

**Keywords:** resilience, brain structure, adversity, cortical thickness, adolescent

## Abstract

Previous research on the neurobiological bases of resilience in youth has largely used categorical definitions of resilience and voxel-based morphometry methods that assess gray matter volume. However, it is important to consider brain structure more broadly as different cortical properties have distinct developmental trajectories. To address these limitations, we used surface-based morphometry and data-driven, continuous resilience scores to examine associations between resilience and cortical structure. Structural MRI data from 286 youths (*M*_age_ = 13.6 years, 51% female) who took part in the European multi-site FemNAT-CD study were pre-processed and analyzed using surface-based morphometry. Continuous resilience scores were derived for each participant based on adversity exposure and levels of psychopathology using the residual regression method. Vertex-wise analyses assessed for correlations between resilience scores and cortical thickness, surface area, gyrification and volume. Resilience scores were positively associated with right lateral occipital surface area and right superior frontal gyrification and negatively correlated with left inferior temporal surface area. Moreover, sex-by-resilience interactions were observed for gyrification in frontal and temporal regions. Our findings extend previous research by revealing that resilience is related to surface area and gyrification in frontal, occipital and temporal regions that are implicated in emotion regulation and face or object recognition.

## Introduction

In 2019, the Global Burden of Disease study estimated that one in seven 10- to 19-year-olds worldwide have at least one mental health condition (Vos *et al*., 2020). Alongside the distress caused to the individual, psychiatric conditions that onset within this developmental period can have negative consequences for their family members and peers (Scott *et al*., 2016). Childhood-onset mental health conditions are also linked to an increased risk of unemployment, as well as mental and physical health problems, in adulthood (Scott *et al*., 2016). Therefore, it is important to understand not only the etiology of childhood-onset mental health difficulties, but also why many young people do not develop such conditions.

An important risk factor for the development of mental health conditions in childhood and adolescence is exposure to adversity or traumatic events (e.g. childhood maltreatment; McLaughlin *et al*., 2012). However, many individuals who experience adversity do not go on to develop mental health problems—instead, they remain resilient (Galatzer-Levy *et al*., 2018). In the current study, resilience is defined as the ability to remain free of significant mental health problems following exposure to adversity (Kalisch *et al*., 2017, 2021). While there are multiple protective ‘resilience factors’ that can promote resilient functioning (Fritz *et al*., 2018), here we focus on a specific aspect of an individual’s neurobiology, namely, their brain structure, in order to better understand potential neurobiological mechanisms of resilience.

Although resilience research can inform preventative interventions for young people who are deemed ‘at risk’ by virtue of being exposed to adversity, a major limitation of this research field is the lack of consensus on how to define and operationalize resilience. For example, some studies have used narrow, categorical definitions of resilience, such as not developing post-traumatic stress disorder (PTSD) following trauma exposure (e.g. Peltonen *et al*., 2014). In contrast, other researchers have taken a broader perspective by assessing how an individual is functioning across different life domains (e.g. educational attainment as well as mental health) to determine whether they can be classified as resilient (e.g. DuMont *et al*., 2007). Nonetheless, in studies that have taken a broader perspective on resilience, there is often no theoretical or data-driven basis for the cut-offs that are used to classify youth as resilient *vs* non-resilient. For example, (DuMont *et al*., 2007) classified youths as resilient if they were functioning well in four or more of the five domains assessed (e.g. graduating from high school and being free of mental health problems). Furthermore, some studies have focused on resilience following childhood maltreatment specifically (e.g. Whittle *et al*., 2013), while others have focused on resilience following a broader variety of traumatic events (e.g. Barzilay *et al*., 2020). Overall, the discrepancies between studies in the way that resilience is defined, operationalized and measured make it difficult to compare them and synthesize their findings.

Over the past two decades, several studies have used neuroimaging techniques to investigate the neural basis of youth resilience (for a systematic review, see Eaton *et al*., 2022). In terms of brain structure, resilience in young people has been not only associated with larger cerebral and cerebellar gray matter volumes (De Bellis *et al*., 2015) and greater prefrontal cortex gray matter volumes (Burt *et al*., 2016), but also smaller total brain volumes (Barzilay *et al*., 2020) in cross-sectional studies. Regarding subcortical structures, a cross-sectional study found that resilience to PTSD was associated with greater left amygdala and right hippocampal volumes (Morey *et al*., 2016), whereas a longitudinal study found that resilience to psychopathology following maltreatment was associated with less pronounced amygdala growth but accelerated hippocampal growth (Whittle *et al*., 2013). It should be noted that all these studies except Whittle *et al*. (2013) took a categorical approach to defining and operationalizing resilience, and this latter study did not focus specifically on resilience but instead on how maltreatment-related changes in brain development might mediate risk for psychopathology.

In a recent study using an overlapping sample to the present one, voxel-based morphometry (VBM) was used to investigate associations between resilience and brain structure in young people and to test for sex differences in those associations (Cornwell *et al*., 2023). Positive correlations between resilience and gray matter volume were identified in several frontal and parietal areas such as the inferior frontal gyrus, along with sex-by-resilience interactions in frontal and temporal areas. In the Cornwell *et al*. (2023) study, a dimensional measure of resilience was derived using the ‘residuals’ approach. The first step in computing resilience scores is to perform a regression analysis to estimate the direction and strength of the relationship between adversity exposure and psychopathology. The discrepancy between this predicted relationship and each individual’s level of psychopathology is then computed to derive ‘resilience residuals’, or resilience scores for each participant. This means that individuals with lower levels of psychopathology than would be expected given their degree of adversity exposure are considered higher in resilience and vice-versa. This analytic approach has been applied successfully to study the genetics of resilience to stressful life events (Amstadter *et al*., 2014) and resilience to other forms of environmental adversity, such as peer victimization and bullying (Bowes *et al*., 2010; Sapouna and Wolke, 2013). In an important paper, Miller-Lewis *et al*. (2013) contrasted four different approaches to operationalizing resilience, including the residuals approach, and found that they yielded broadly similar findings in terms of identifying associations between child mental health resilience and child and family ‘resource’ factors such as child self-esteem or a positive child–parent relationship. However, they noted that the residuals approach was statistically more powerful than other approaches and allowed them to include their full sample in their analyses, rather than focusing on subsamples (i.e. the minority of children who experienced both high adversity and low levels of mental health difficulties within person-centered analyses).

As was the case for the Cornwell *et al*. (2023) study, most studies investigating the structural brain basis of resilience have used VBM methods which assess gray matter volume across the entire brain. However, the cortex can be described by several properties including cortical thickness, surface area and gyrification (i.e. cortical folding). Surface-based morphometry (SBM) distinguishes between these different aspects of cortical structure, which have distinct genetic underpinnings and developmental trajectories (Panizzon *et al*., 2009; Raznahan *et al*., 2011; Li *et al*., 2014), and can thus provide a more fine-grained understanding of brain structure than VBM. Therefore, it is important that we study these cortical properties separately when using youth samples to disentangle which specific cortical properties are driving the effects on (gray matter) volume identified in previous resilience research, and whether novel associations could be detected when using more sensitive and specific measures (e.g. cortical thickness or surface area).

There has also been limited consideration of possible sex differences in the neurobiological bases of resilience in previous research. Research has demonstrated sex differences in brain structure during adolescence in typically developing samples (Lenroot and Giedd, 2010; Paus *et al*., 2017), in the development of different cortical properties (Giedd, 2004; Raznahan *et al*., 2011; Lyall *et al*., 2015), and in the neurobiology of psychopathology (e.g. Helpman *et al*., 2017; Smaragdi *et al*., 2017). It is also well-established that there are marked sex differences in the prevalence of some forms of psychopathology, such as depression (Hankin *et al*., 1998). Therefore, sex differences in the relationship between resilience and cortical structure might be expected. However, previous studies have been too small to reliably test for sex differences in the relationship between resilience and brain structure or have used categorical measures of resilience which do not lend themselves well to testing for such interactions.

Thus, our primary aim was to investigate associations between resilience and cortical structure and subcortical volumes in youth. To achieve this, we used data-driven, continuous resilience scores derived in a previous study using data on lifetime adversity exposure and psychopathology (Cornwell *et al*., 2023) and related these scores to cortical volume, thickness, surface area and gyrification in a large European sample of youth aged between 9 and 18 years. Furthermore, based on the findings of our recent systematic review of neuroimaging studies of resilience in youth (Eaton *et al*., 2022) and narrative reviews of resilience studies in adults (e.g. Bolsinger *et al*., 2018), we also used FreeSurfer’s subcortical pipeline to estimate amygdala and hippocampal volumes and relate these to resilience scores. Our secondary aim was to test for sex differences in the relationship between resilience and cortical structure and subcortical volumes. Based on previous research, we hypothesized that resilience would be positively correlated with cortical volume, thickness and surface area in frontal and parietal regions, and that sex differences in the associations between resilience and cortical structure would be observed. We also predicted that resilience would be positively correlated with amygdala and hippocampal volumes. As no resilience studies have focused on cortical gyrification, we did not formulate specific hypotheses about this SBM metric.

## Methods

The current study used secondary data from the FemNAT-CD study, which aimed to investigate sex differences in Conduct Disorder (CD; Freitag *et al*., 2018). Methods relevant to the current study are provided below. Further details about the FemNAT-CD study, including inclusion and exclusion criteria, recruitment strategies and assessment procedures, can be found in Konrad *et al*. (2022). The current study was approved by the University of Bath’s Psychology Research Ethics Committee (Ref: 18–322). Participants and their parents or carers gave informed consent/assent to take part in the FemNAT-CD study.

### Participants

**Fig. 1.**
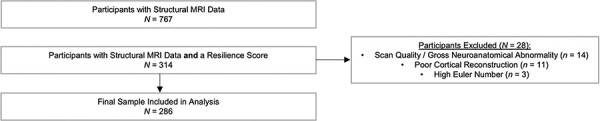
A flowchart detailing the sample selection process for the current study.

Figure 1 shows the sample selection process. In brief, 767 youths aged between 9 and 18 years from five sites across Europe took part in the neuroimaging work package. Of these, 286 (37%) were eligible for inclusion in the current study based on having a resilience score and useable structural MRI data following detailed quality control checks (including reviewing cortical segmentations)—the majority of those excluded had missing data on one or more of the adversity measures used to derive the resilience scores. Figure S1 shows the number of participants who were included from each site. It should be noted that the 286 participants included here are a subsample of the 298 included in our previous VBM study (Cornwell *et al*., 2023)—the difference in numbers is explained by exclusions based on issues with cortical segmentation.

Due to the original aims of the FemNAT-CD study, 26% of the present sample (75 participants) had a research diagnosis of CD, while the other 211 participants (74%) were free of current Axis I disorders and past CD, Oppositional Defiant Disorder (ODD) and Attention-Deficit/Hyperactivity Disorder (ADHD). Participants with CD were allowed to have comorbid psychiatric diagnoses, except for autism spectrum disorders, bipolar disorder/mania or schizophrenia, which were exclusion criteria for both groups (Konrad *et al*., 2022).

### Measures

Diagnoses of CD and other psychiatric disorders were made using the Kiddie-Schedule for Affective Disorders and Schizophrenia (K-SADS-PL; Kaufman *et al*., 1997), a diagnostic interview completed separately by participants and their parents/carers (see Supplement 1 for inter-rater reliability data). Sex was self-reported; we did not ask about the participants’ gender identity. IQ was estimated using the vocabulary and matrix reasoning subscales of the Wechsler Abbreviated Scale of Intelligence (WASI; Wechsler, 1999) at the UK sites or the Wechsler Intelligence Scale for Children-IV (WISC-IV; Wechsler, 2003) at all other sites. Pubertal status was measured using the self-report Pubertal Development Scale (PDS; Petersen *et al*., 1988).

### Resilience scores

The first step in the residual regression approach was to perform a regression analysis to estimate the direction and strength of the relationship between adversity exposure and psychopathology. We then calculated the discrepancy between this predicted relationship and each individual’s level of psychopathology to derive ‘resilience residuals’, or resilience scores for each individual. Youths with positive residual scores (i.e. those falling above the regression line that was fitted) are thus considered to show ‘better than expected’ mental health or lower levels of psychopathology than would be expected given their degree of adversity exposure and viewed as being higher in resilience or resilient functioning at that point in time. Youths with negative residual scores (i.e. those falling below the regression line) are considered to show ‘worse than expected’ mental health or higher levels of psychopathology than would be expected given their degree of adversity exposure. These individuals are viewed as being lower in resilience or resilient functioning, with strongly negative scores observed in those with very high levels of psychopathology in the context of low or negligible adversity exposure. [This process of deriving resilience scores is described in more detail in van Harmelen *et al*. (2017) and Ioannidis *et al*. (2020), and a helpful visual representation of the residual regression approach is provided in these papers, together with a critique of the residuals approach and a comparison with other approaches to defining and measuring resilience.]

The resilience scores used here were derived in a separate study (Cornwell *et al*., 2023). Briefly, two principal axis factor analyses were run as a method of data reduction. The first factor analysis was run on data from two interviews and a questionnaire measuring lifetime exposure to adversity and traumatic events (e.g. witnessing a violent crime and physical abuse): the parent-report Children’s Bad Experiences interview (Arseneault *et al*., 2011), the PTSD subsection of the K-SADS-PL (conducted with parents/carers and children in separate confidential interviews) and the child-report Childhood Experience of Care and Abuse questionnaire (CECA-Q; Bifulco *et al*., 2005; see Supplement 1 for reliability data). The second factor analysis was run on psychopathology data acquired using the K-SADS-PL (conducted with parents/carers and children) and the parent-report Child Behavior Checklist (CBCL; Achenbach, 1991), capturing current and lifetime symptoms of internalizing and externalizng disorders including affective, anxiety and disruptive behavior disorders (see Supplement 1 for reliability data).

Factor scores were then weighted by the variance they individually explained, range normalized between 1 and −1 to ensure that they were of comparable magnitude and aggregated using the median operator (separately for adversity exposure and psychopathology factors). The aggregate adversity exposure and lifetime psychopathology scores were entered into a regression model. An individual resilience score between 1 and −1 was calculated for each participant by calculating the individual distance from the regression line along the psychopathology dimension.

Youths who were lower in psychopathology than expected given their degree of adversity exposure had higher resilience scores and vice-versa. Figures S2 and S3 display the relationships between adversity exposure and psychopathology, adversity exposure and resilience scores and psychopathology and resilience scores in the current sample by diagnostic group and sex, respectively.

### MRI data acquisition

Structural MRI data were obtained at five different sites across Europe using Siemens 3 T (Tim-Trio and Prisma) or Philips 3 T (Achieva) scanners. T1-weighted scans were acquired using magnetization-prepared rapid acquisition gradient-echo sequences. The acquisition parameters were harmonized across sites under the supervision of an MR physicist and each site underwent site qualification procedures before starting data collection, which included scanning phantoms and checking for hardware problems (further details are given in the Supplementary Materials). Image quality was assessed by a trained radiographer immediately after each scan and, if necessary, the scan was repeated.

### Image processing

First, the quality of all eligible T1-weighted scans was inspected using a published protocol (Backhausen *et al*., 2016) in MRIcron (https://www.nitrc.org/projects/mricron/). Each scan was rated by two independent raters blind to participant’s status based on the following four criteria: image sharpness, ringing and contrast-to-noise ratio of subcortical structures and gray and white matter. Scans rated as fails (including scans with gross neuroanatomical abnormalities; *n *= 14) were excluded from further analysis.

To ensure consistency with previous FemNAT-CD papers (e.g. Smaragdi *et al*., 2017), FreeSurfer v5.3.0 (https://surfer.nmr.mgh.harvard.edu/) was used to estimate cortical volume, thickness, surface area and gyrification at each vertex. The cortical reconstruction process involves segmenting the white matter and identifying the white matter–gray matter and gray matter–cerebrospinal fluid interfaces to create the pial surface (see Fischl, 2012). For cortical volume, cortical thickness and surface area, smoothing was performed with a 5 mm kernel at full width/half maximum. The gyrification measures were not smoothed because the local gyrification index (lGI) is inherently smooth (Schaer *et al*., 2013). Each participant’s cortical segmentation was visually inspected and, if necessary, manual edits were made to the white matter or pial boundaries. These edits, which were done blind to group status, could involve adding or deleting white or gray matter and setting intensity normalization control points. In total, 254 scans were manually edited at least once, although it should be noted that manual edits were performed in line with best practice guidelines outlined by the FreeSurfer development team (Draudt, 2022) and were kept to a minimum. Finally, hippocampal and amygdala volumes were estimated using FreeSurfer’s automatic segmentation pipeline (Fischl *et al*., 2002).

### Statistical analyses

We performed whole-brain vertex-wise analyses in FreeSurfer. We employed separate General Linear Models (GLMs) for cortical volume, thickness, surface area and lGI to explore correlations between resilience scores and cortical structure. A resilience score-by-sex interaction term was generated by multiplying demeaned resilience scores by the dichotomous sex variable in SPSS Version 26. This was done to test for sex differences in the direction or strength of the associations. All statistical models included sex, age, diagnostic group (CD or healthy control) and scanner site (coded using the ‘one-hot encoding’ approach; Hancock and Khoshgoftaar, 2020) as covariates of no interest. Estimated total intracranial volume (eTIV) was calculated using FreeSurfer and included as an additional covariate in the cortical volume, surface area and gyrification analyses to control for variability in overall brain size. However, cortical thickness does not scale linearly with global brain volumes so eTIV was not included as a covariate in this analysis.

Further GLMs testing for associations between resilience scores and hippocampal and amygdala volumes and sex-by-resilience score interactions on the volumes of these regions were run in R v4.1.0 (https://www.r-project.org/). Again, sex, age, diagnostic group and scanner site (dummy coded), and eTIV were included as covariates of no interest. Four GLMs were fitted, one for each hemisphere for both regions. All analytical decisions were made to maintain consistency between the whole-brain vertex-wise and subcortical analyses.

For our cortical structure analyses, we performed cluster-wise multiple comparisons corrections using Monte Carlo z-field simulations (Hagler *et al*., 2006) and report clusters with vertex-wise and cluster-wise thresholds of *P *< 0.05. To correct for multiple comparisons in our subcortical analyses, we applied a False-Discovery-Rate correction (*q *= 0.05; Benjamini and Hochberg, 1995).

## Results

### Demographic and clinical characteristics

Participants included in the analysis were aged 9–18 years (*M *= 13.60, *SD *= 2.55), 51% were female, and their mean IQ was 103.65 (*SD *= 12.12). Resilience scores ranged from 0.22 to −0.59 (where positive scores reflect higher resilience). Current CD symptoms ranged from 0 to 11 (*M *= 1.16, *SD *= 2.17), which reflected the inclusion of participants with CD and healthy controls. The number of traumatic events experienced during the lifetime, based on the K-SADS-PL PTSD screen, ranged from 0 to 7 (*M *= 1.24, *SD *= 1.35), which was similar to the range observed in the full sample (0–8). The variance in CD symptoms was also highly comparable in the present subsample compared to the full FemNAT-CD sample (0–11 and 0–13 CD symptoms, respectively). In terms of other lifetime DSM-IV-TR diagnoses, 65 (23%) participants had a diagnosis of ODD, 44 (15%) had a diagnosis of ADHD, 18 (6%) had a diagnosis of major depressive disorder and 19 (7%) had a diagnosis of anxiety disorders. These individuals were all in the CD group because having a current psychiatric diagnosis was an exclusion criterion for the healthy control group.

### Cortical analyses

#### Correlations with resilience scores

**Fig. 2.**
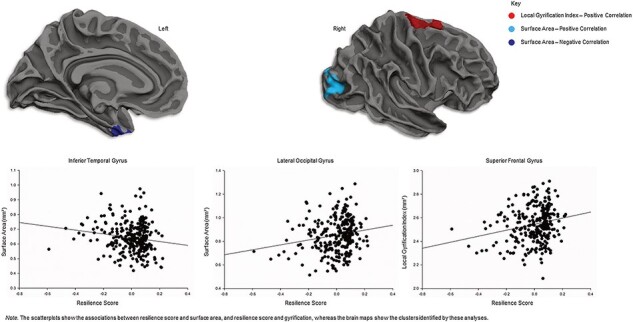
Correlations between resilience scores and cortical structure. Positive correlations were observed between resilience and surface area in the lateral occipital gyrus (**a**) and gyrification in the superior frontal gyrus (**b**). A negative correlation between resilience and surface area was observed in the inferior temporal gyrus (**c**).

Across the entire sample, resilience scores were positively correlated with surface area in the right lateral occipital gyrus, and negatively correlated with surface area in the left inferior temporal gyrus (see Table 1 and Figure 2). Resilience scores were also positively correlated with superior frontal gyrification (Figure 2). There were no significant correlations between resilience scores and cortical thickness or cortical volume.

**Table 1.** Correlations between resilience scores and cortical structure and sex-by-resilience score interactions

						MNI coordinates				
	Brain region	BA	Hemisphere	NVtxs	Size (mm^2^)	*x*	*y*	*z*	Max	CWP
Cortical surface area										
Overall positive correlation	Lateral occipital gyrus	18	R	1184	954.41	31	−92	1	2.41	0.003
Overall negative correlation	Inferior temporal gyrus	20	L	707	724.73	−30	−4	−44	−4.22	0.030
Local gyrification index										
Overall positive correlation	Superior frontal gyrus	6	R	1350	802.35	17	5	65	3.11	0.007
Females positive, males negative	Rostral anterior cingulate cortex	32	L	1426	1126.72	−10	42	9	−2.22	<0.001
	Middle temporal gyrus	21	L	1077	847.43	−55	−12	−18	−3.97	0.002
	Medial orbitofrontal cortex	11	R	561	638.76	5	58	−21	−2.15	0.031

BA, Brodmann area; CWP, cluster-wise *P*-value; L, left; Max, maximum −log10 (*P*-value) in the cluster; MNI, Montreal Neurological Institute; NVtxs, number of vertices; R, right.

#### Sex-by-resilience interactions

**Fig. 3.**
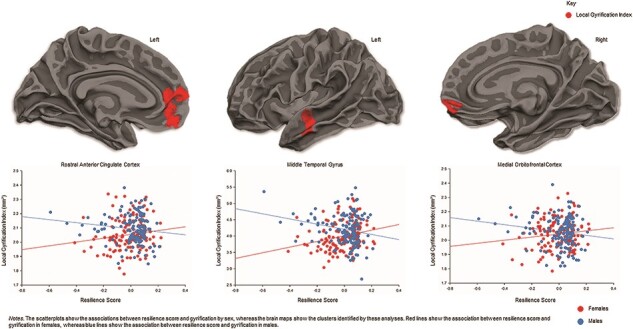
Sex-by-resilience interactions for gyrification. Resilience scores were positively correlated with gyrification in the rostral anterior cingulate cortex, middle temporal gyrus and medial orbitofrontal cortex in female youth, but negatively correlated in male youth.

Sex-by-resilience score interactions were observed for gyrification (see Table 1 and Figure 3). Specifically, resilience scores were positively correlated with gyrification in the left rostral anterior cingulate cortex, right medial orbitofrontal cortex and left middle temporal gyrus in females. In contrast, negative correlations with resilience scores were observed in all three of these regions in males. There were no sex-by-resilience interactions for cortical thickness, surface area or volume.

### Subcortical analyses

Resilience scores were not significantly correlated with hippocampal and amygdala volumes. A weak sex-by-resilience interaction was detected in the left amygdala (*P *= 0.048, uncorrected), which was driven by a positive association between resilience scores and amygdala volume in females but not males; however, this did not survive correction for multiple comparisons. There were no other significant sex-by-resilience score interactions.

### Sensitivity analyses

Given that there were group differences and sex-by-group interactions in IQ (Table S2), we ran an additional analysis controlling for IQ. The cortical structure results reported above remained significant when controlling for IQ (Table S3). Again, there were no significant findings for cortical thickness or cortical volume in this analysis.

Furthermore, given that there were group differences in resilience scores (Table S2), we ran two additional sensitivity analyses—one including just healthy controls and one including just CD participants. Very similar results were obtained when considering each group separately (see Tables S4 and S5), except for an additional negative correlation between resilience scores and left superior frontal surface area in the healthy control group. Furthermore, the correlation between resilience scores and right superior frontal gyrification was negative and was observed in a different part of the right superior frontal gyrus, namely, the dorsomedial prefrontal cortex. In the healthy control group, there were no significant findings for cortical volume, whereas in the CD group, there were no findings for cortical surface area or cortical volume. These results overall suggest that the resilience effects detected in the full sample were not driven by group differences in cortical structure (or IQ).

Finally, to focus on a more homogenous subsample in terms of developmental stage, we repeated the analyses including only the participants who had started pubertal development, based on the self-report PDS (*n *= 218). Findings were broadly similar to those obtained in the full sample, although an additional positive correlation between resilience and left pericalcarine cortical volume was detected (Table S6).

## Discussion

This study investigated whether resilience in young people is related to differences in cortical structure and hippocampal and amygdala volumes. We found that resilience was positively associated with surface area in the right lateral occipital gyrus, and positively associated with right superior frontal gyrification. We also found that resilience was negatively associated with left inferior temporal gyrus surface area. However, contrary to our predictions, resilience was not related to cortical structure in parietal regions or the volume of key subcortical regions that are sensitive to adversity or maltreatment or implicated in resilience (i.e. the hippocampus and amygdala).

Our second aim was to test for sex-by-resilience interactions on cortical structure. In line with our hypothesis, the associations between resilience and gyrification in frontal and temporal regions differed between female and male youth. In all three regions, resilience was positively correlated with gyrification in female youth, but negatively correlated in male youth. Interestingly, no correlations with resilience or sex-by-resilience interactions were observed for cortical volume or thickness. Overall, our results provide preliminary evidence that alterations in surface area and gyrification might be driving the volumetric effects in certain regions that have been observed in previous VBM studies of resilience and earlier structural MRI studies that used brain parcellation methods, although associations between resilience and cortical volume were not observed in the current study.

Although the findings of our previous VBM study (Cornwell *et al*., 2023) were specific to gray matter volume, there was some convergence in the brain regions related to youth resilience across both studies. Firstly, resilience was associated with cortical structure in areas of the prefrontal cortex (including the superior frontal and middle and inferior frontal gyri), an area of the brain that plays a critical role in emotion processing and regulation (Dixon *et al*., 2017). Furthermore, a sex-by-resilience score interaction in the middle temporal gyrus was uncovered in both studies. However, it should be noted that these interactions were in the opposite direction, although this could be explained by the fact that one finding was for gray matter volume, whilst the other was for gyrification. This demonstrates the importance of looking at each cortical property separately. In terms of findings in subcortical regions, contrary to our hypotheses, we found no correlations between resilience and hippocampus or amygdala volumes.

In the current study, we identified a positive correlation between resilience scores and surface area in the right lateral occipital gyrus. This is part of the secondary or extrastriate visual cortex and is involved in object and face recognition (Grill-Spector and Malach, 2004), as well as motion and color perception (Strotzer, 2009). Although this region has not been identified in previous structural MRI studies of resilience, Teicher and Samson (2016) reviewed evidence demonstrating maltreatment-related effects on visual cortex structure in children and adults. This could suggest that young people who remain resilient following maltreatment (or adversity exposure more generally) do not show maltreatment-related changes in the visual cortex—or might even show compensatory changes in this region. Resilience was also positively correlated with gyrification in the superior frontal gyrus, an area involved in motor learning and planning (Strotzer, 2009). Interestingly, Burt *et al*. (2016) found that superior frontal gyrus gray matter volume was greater in resilient adolescents compared to other groups (i.e. adversity-exposed adolescents with impaired functioning and non-exposed adolescents). Of note, previous research in normative samples has reported strong associations between cortical volume and surface area (e.g. Winkler *et al*., 2010). Our findings and those of Burt *et al*. (2016) suggest that the superior frontal gyrus is an important region in terms of youth resilience.

We also observed a negative correlation between resilience and surface area in the left inferior temporal gyrus. The inferior temporal gyrus is implicated in object recognition (Herath *et al*., 2001) and impulsivity (Li and Kong, 2017). Although the inferior temporal gyrus has not been implicated in youth resilience previously, a recent study found trait resilience was positively correlated with cortical thickness in this region in adults (Kahl *et al*., 2020). Overall, these findings suggest that morphological alterations (e.g. reduced surface area or increased cortical thickness) in the inferior temporal gyrus may confer resilience. However, this also demonstrates that sample characteristics (e.g. age), the definition of resilience adopted and the structural metric measured may impact study findings. This adds weight to the argument that resilience should be defined consistently across studies, and that different cortical properties should be distinguished because they may be related to resilience in different ways.

We also identified sex-by-resilience interactions on gyrification in the rostral anterior cingulate and medial orbitofrontal cortices and middle temporal gyrus. In all three regions, resilience was positively correlated with gyrification in females, but negatively correlated in males. In our recent VBM study based on a largely overlapping sample, we found that resilience was positively associated with gray matter volume in the middle temporal gyrus in male youth, but negatively correlated with gray matter volume in this region in female youth (Cornwell *et al*., 2023). The differences between the cortical gyrification and gray matter volume findings in our SBM and VBM studies, respectively, are in line with a recent study, which found only partial overlap between alterations in gyrification and cortical volume in adults with schizophrenia (Spalthoff *et al*., 2018). Furthermore, a recent review of the emotion-related functions of the prefrontal cortex found that the pregenual anterior cingulate cortex is involved in the evaluation of interoceptive signals, while the medial orbitofrontal cortex is involved in the appraisal of internal mental simulations (Dixon *et al*., 2017). In a recent narrative review of adult studies of resilience (Bolsinger *et al*., 2018), reduced anterior cingulate cortex volumes were found to be associated with vulnerability to the mental health impact of traumatic events (i.e. the opposite of resilience). Additionally, in the aforementioned review by Teicher and Samson (2016), the anterior cingulate and orbitofrontal cortices were identified as particularly susceptible to maltreatment. Taken together, these results suggest that structural alterations in areas involved in emotion generation and regulation may be markers of resilience *vs* vulnerability to adversity or trauma. This fits with previous research that has identified emotion regulation ability as a key resilience factor (Fritz *et al*., 2018). However, the sex differences in gyrification in these areas identified in the current study should be explored in future research (and related to neurocognitive or functional brain outcomes).

Our sensitivity analyses showed that the findings largely remained significant when controlling for IQ or considering each diagnostic group separately. This suggests that our results are not confounded by IQ or group differences in resilience scores. Although we controlled for age in our analyses, we acknowledge that a significant proportion of children in the sample were younger than the average age of onset for many psychiatric disorders (especially depression; Thapar *et al*., 2012), and that each participant’s level of resilience may fluctuate over their lifetime. It has also been proposed that for some children, the impact of adversity is not immediately apparent but it may lead to changes in brain functioning that confer vulnerability to future stressors, known as ‘latent vulnerability’ (McCrory and Viding, 2015), or that there may be distinctive neurobiological processes that promote resilient functioning depending on the type and timing of adverse experiences, as well as the timing of the assessment of psychopathology (Ioannidis *et al*., 2020). Our results also held when confining the analysis to a subsample who had reached the peak risk period for developing psychiatric disorders (i.e. puberty); however, it remains to be determined whether the association between resilience and brain structure differs according to the timing of the adversity as we did not have fine-grained information about this.

### Strengths and limitations

Our study had several strengths. To our knowledge, this was the first study to investigate associations between youth resilience and cortical structure using SBM, which separates composite measures of volume into distinct cortical properties, each with unique developmental trajectories (Raznahan *et al*., 2011) and genetic underpinnings (Panizzon *et al*., 2009). There is also evidence that SBM is more sensitive than VBM in detecting associations between psychopathology and brain structure (Palaniyappan and Liddle, 2012), so the same may be true for resilience. We adopted rigorous quality control procedures, including checking for segmentation errors, and manually editing the white matter and pial boundaries where necessary. Furthermore, we used a continuous measure of resilience that allowed us to explore associations between resilience and cortical structure without imposing narrow or arbitrary cut-offs in terms of who should be classified as resilient (unlike many previous studies that have used categorical approaches, such as not developing PTSD following childhood maltreatment). Our continuous resilience scores were also derived using data on participants’ exposure to a range of adversities, some more normative (e.g. poor relationships with parents) and others more severe (e.g. physical abuse), and various forms of psychopathology (covering a range of internalizing and externalizing disorders). A similar approach has been adopted by researchers studying the genetics of resilience to stressful life events (Amstadter *et al*., 2014), and resilience to peer victimization and bullying (Bowes *et al*., 2010; Sapouna and Wolke, 2013). It has also been shown that the residuals approach yields congruent findings with other approaches to defining resilience but is more powerful because it avoids focusing on the (typically) small subset of young people who are exposed to adversity but who remain low in psychopathology (Miller-Lewis *et al*., 2013). Furthermore, we also took a multi-informant approach when assessing both adversities (i.e. we asked both the participants and their parents/caregivers about the presence of potentially traumatic events) and symptoms of psychopathology, which mitigates against problems of shared method variance. Finally, adopting a continuous approach to resilience enabled us to test for sex differences in the association between resilience and cortical structure for the first time in the youth neuroimaging literature.

However, the study also had several limitations. First, the FemNAT-CD study was originally designed to investigate sex differences in CD and therefore the sample was not representative of the general population—it was comprised of a mix of ‘super-well’ healthy controls without any diagnosable disorders and youth with CD, many of whom had comorbid disorders such as ADHD or major depressive disorder. This means that factors related to externalizing disorders (both current and lifetime symptoms) were the most important contributors to the psychopathology factor scores that were used to compute each participant’s resilience score. The resilience scores and SBM findings may have been different if we had recruited a psychiatric control group without CD as well as a healthy control group without any disorders—or conducted this study using a fully representative, population-based sample. The unique characteristics of the sample, and the fact that many participants were recruited on the basis of having CD, may also have led to the weak correlation between adversity and psychopathology observed here (*r*^2^ = 0.13). This is potentially problematic in implementing the residuals approach although it should be noted that the relationship between these variables was still in the expected direction (i.e. positive). Additionally, the structural MRI data were collected using different scanners at different sites. Although this was partly controlled for by ensuring that the acquisition parameters were harmonized across sites and by including scanner site as a covariate, it may have introduced some noise into the data. There were group differences in resilience scores, such that the CD youths tended to have lower scores and the range of resilience scores observed in this group was larger. Moreover, there was a sex-by-group interaction for age and a main effect of group on IQ (such that the CD group had a lower average IQ). It is possible that some of our findings were partially explained by these factors, although this should have been mitigated to an extent by controlling for these variables in our analyses and performing sensitivity analyses in each group separately. In addition, we acknowledge that resilience is not static and therefore an individual’s level of resilience cannot be determined based on data from a single time point (Ioannidis *et al*., 2020). Therefore, although we controlled for age effects in our SBM analyses, a substantial proportion of the children were younger than the average age of onset for most psychiatric disorders (Paus *et al*., 2008) so they may not prove to be resilient over the longer term. Consequently, it has been argued that a more appropriate term to use in this context is ‘resilient functioning’ rather than resilience (Ioannidis *et al*., 2020). Furthermore, the number of CD participants included in the sensitivity analysis was small, particularly when considering the sex-by-resilience interaction analysis—which may have impacted the statistical power of these analyses. Finally, we acknowledge that our study had a cross-sectional rather than a longitudinal design, and this prevented us from ascertaining whether the observed differences in cortical structure were present prior to adversity exposure, and therefore represent more ‘trait-like’ resilience effects, or whether they emerged following adversity exposure in a manner that is more consistent with contemporary outcome or process approaches to resilience (Eaton *et al*., 2022). Future studies should adopt prospective longitudinal designs to investigate whether the present associations between resilient functioning and cortical structure reflect pre-existing differences or adaptations in brain structure that emerge following childhood adversity or trauma—which would fit better with the concept of resilience as an emergent process rather than an intrinsic trait or dispositional characteristic of the individual.

## Conclusions

Using a data-driven, dimensional measure of resilience, we found that resilience in youth was linked to greater surface area and gyrification in occipital and frontal regions, respectively, and lower surface area in temporal regions. However, resilience was not associated with cortical thickness, cortical volume or amygdala or hippocampal volumes after correcting for multiple comparisons. We also identified sex-by-resilience interactions on gyrification in key frontal and temporal regions such as the rostral anterior cingulate and medial orbitofrontal cortices. Our study highlights the importance of using SBM methods to investigate associations between resilience and different aspects of cortical structure, rather than focusing on gray matter volume alone, as earlier studies have done. Our findings also provide preliminary evidence for sex differences in the neurobiological basis of resilience and highlight the need to take account of sex or gender in future research. Overall, this study provides evidence that youth resilience may be related to structural changes in brain regions involved in emotion regulation, object/face recognition and impulsivity.

## Supplementary Material

nsae006_Supp

## Data Availability

No new data were generated or analyzed in support of this research. Data supporting this study are not publicly available but can be requested from the FemNAT-CD Steering Committee, which is chaired by Professor Christine Freitag: C.Freitag@em.uni-frankfurt.de
