# Supplementary material for: Identifying cortical structure markers of resilience to adversity in young people using surface-based morphometry
Source: Soc Cogn Affect Neurosci. 2024 Jan 27;19(1):nsae006. doi: 10.1093/scan/nsae006 (PMC10868125; doi:10.1093/scan/nsae006)
Supplement: nsae006_Supp [file nsae006_supp.zip › scan-23-153-File006.docx]

## Supplementary Materials to Cornwell et al. *Identifying Cortical Structure Markers of Resilience to Adversity in Young People using Surface-Based Morphometry.*

**Figure S1**

*Number of Participants from Each Site Included in the Final Analysis (N=286)*


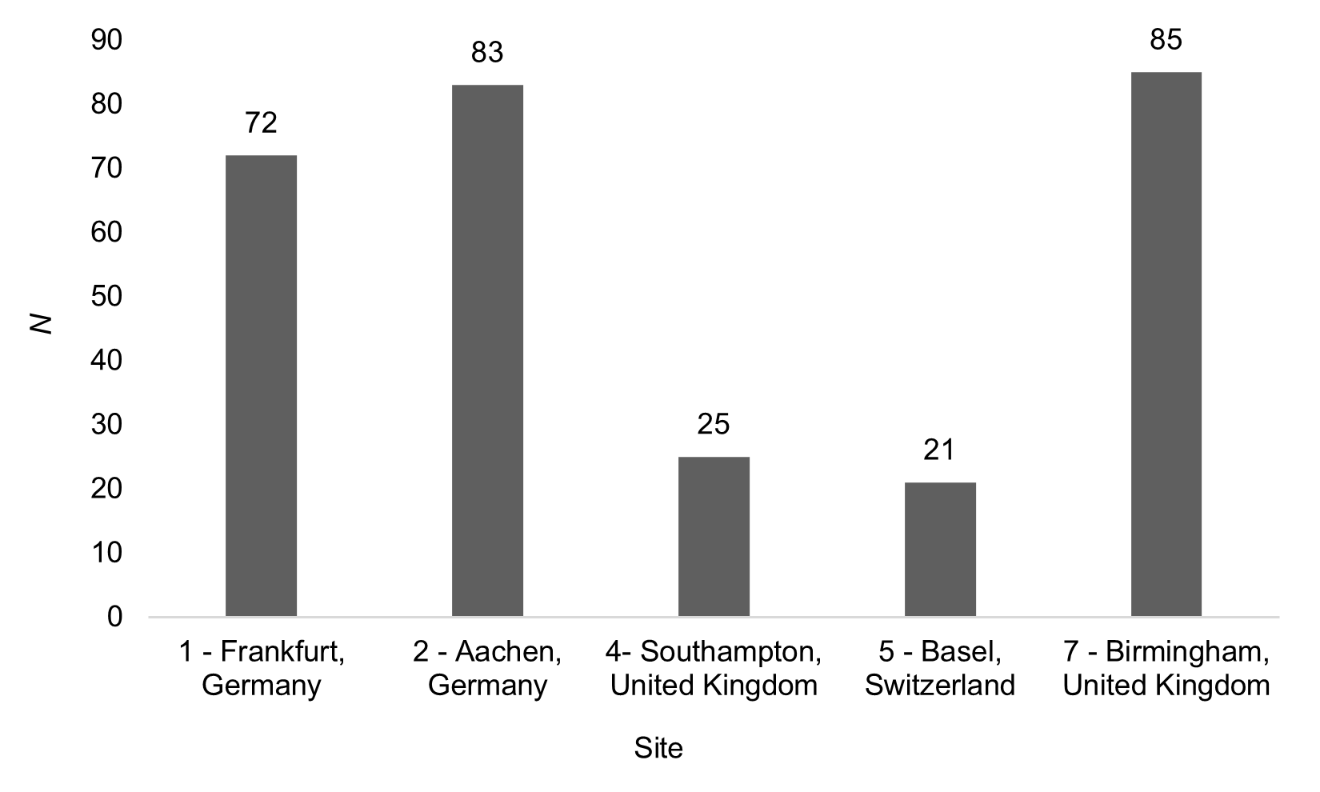


**Supplement 1 – Reliability**

- K-SADS-PL: Inter-rater reliability of current CD diagnoses and other comorbid diagnoses (e.g., Oppositional Defiant Disorder, Attention-Deficit/Hyperactivity Disorder, and Major Depressive Disorder) was assessed across all FemNAT-CD sites. All values fell within the range of strong to almost perfect agreement (Cohen’s kappas ≥ 0.84; McHugh, 2012).
- CECA-Q: Bifulco et al. (2005) reported that the antipathy and neglect dimensions of the CECA-Q had good reliability (Cronbach’s alphas ≥ 0.80).
- CBCL: Achenbach and Rescorla (2001) reported that the internalising problems, externalising problems, and total problems scores had excellent reliability (Cronbach’s alphas ≥ 0.90).

**Figure S2**

*Scatterplots Illustrating the Relationships Between: (a) Adversity Exposure and Psychopathology, (b) Adversity Exposure and Resilience, and (c) Psychopathology and Resilience, with CD and healthy control participants shown in different colors.*

**
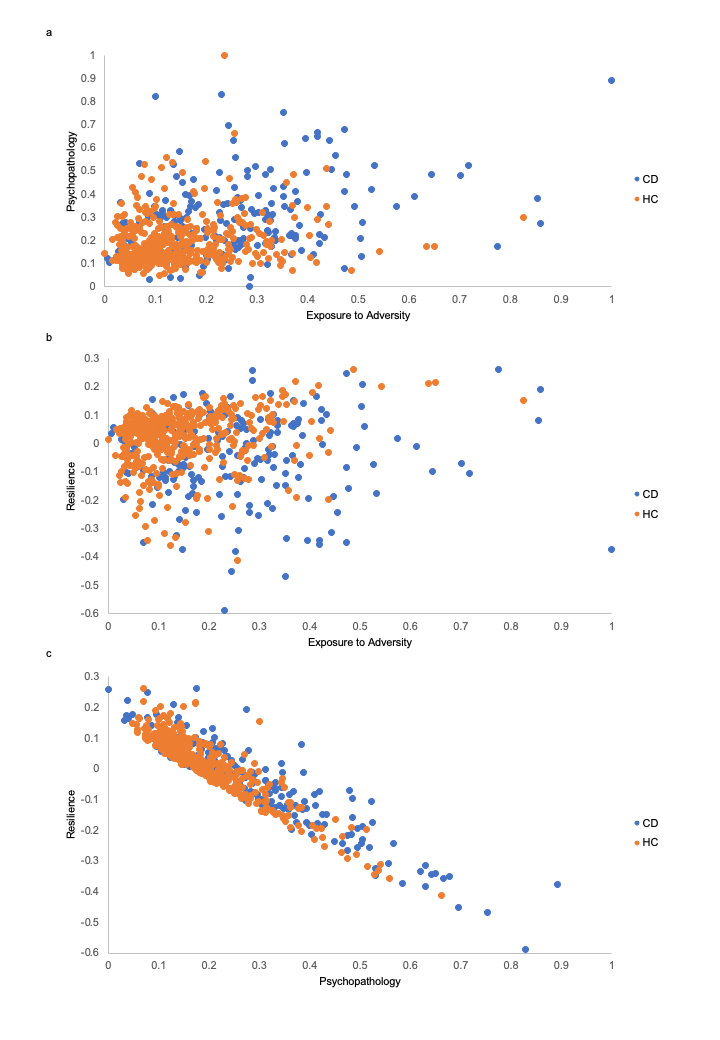
**

*Notes.* CD, Conduct Disorder; HC, Healthy Controls. Each symbol represents a different individual.

**Figure S3**

*Scatterplots Illustrating the Relationships Between: (a) Adversity Exposure and Psychopathology, (b) Adversity Exposure and Resilience, and (c) Psychopathology and Resilience, with male and female participants shown in different colors.*


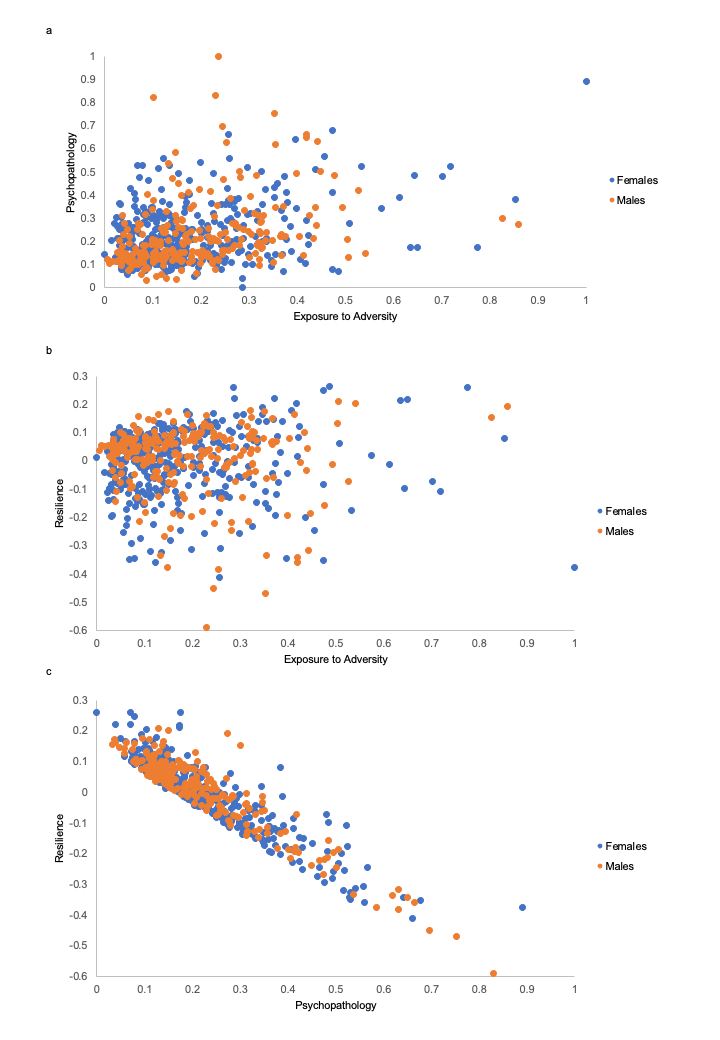


*Notes.* Each symbol represents a different individual.

**Table S1**

|  | Site 1 - Frankfurt |  | Site 2 - Aachen |  | Site 4 - Southampton |  | Site 5 - Basel |  | Site 7 - Birmingham |
| --- | --- | --- | --- | --- | --- | --- | --- | --- | --- |
| Scanner | Siemens Magnetom Tim Trio |  | Siemens Magnetom Prisma |  | Siemens Magnetom Tim Trio |  | Siemens Magnetom Prisma |  | Philips Achieva |
| Software version | Syngo MR A35 |  | Syngo MR D13D |  | Syngo MR B17 |  | Syngo MR D13D |  | Version 3.2.6.1 |
| Head coil | 8-channel |  | 20-channel |  | 32-channel |  | 20-channel |  | 32-channel |
| Number of slices | 192 |  | 192 |  | 192 |  | 192 |  | 192 |
| Voxel size | 1 x 1 x 1 mm |  | 1 x 1 x 1 mm |  | 1 x 1 x 1 mm |  | 1 x 1 x 1 mm |  | 1 x 1 x 1 mm |
| Repetition time (TR) | 1900ms |  | 1900ms |  | 1900ms |  | 1900ms |  | 1900ms |
| Echo time (TE) | 2.74ms |  | 3.42ms |  | 4.1ms |  | 3.42ms |  | 3.7ms |
| Inversion time (TI) | 900ms |  | 900ms |  | 900ms |  | 900ms |  | 900ms |
| Field of view | 256mm |  | 256mm |  | 256mm |  | 256mm |  | 256mm |
| Flip angle | 9° |  | 9° |  | 9° |  | 9° |  | 9° |

*Scanner Type and Acquisition Parameters, by Site*

**Supplement 2 – Site Qualification Procedures**

To ensure comparability of magnetic resonance imaging (MRI) data acquisition across sites, all sites collecting MRI data underwent site qualification procedures prior to beginning data collection. The following three quality assurance checks were completed:

1. The first involved scanning an American College of Radiology (ACR) phantom (Chen et al., 2004). This first check was designed to assess structural MRI sequences.
2. The second quality assurance check assessed scanning stability during functional MRI data acquisition and involved scanning a Functional Biomedical Infrastructure Research Network (FBIRN) phantom (Glover et al., 2012).
3. The final quality assurance check involved scanning a human volunteer using a FemNAT-CD compliant imaging protocol.

Once all three quality assurance checks were performed, the resulting images were reviewed by an MRI physicist at the University of Birmingham (Dr. Ali Chowdhury). Feedback was provided by the physicist on any adjustments that needed to be made to the scanning parameters. All sites had to pass the three quality assurance checks prior to collecting any MRI data.

**Table S2**

*Demographic and Clinical Characteristics of the Participants Included in the Analysis Split by Sex and Diagnostic Group (N=286)*

|  | Female (*n*=147) | | | | | Male (*n*=139) | | | |  | |  | |  |
| --- | --- | --- | --- | --- | --- | --- | --- | --- | --- | --- | --- | --- | --- | --- |
|  | CD (*n*=22) | | | HC (*n*=125) | | CD (*n*=53) | | HC (*n*=86) | |  | |  | |  |
| Characteristic | | *M (SD)* | *M (SD)* | | *M (SD)* | | *M (SD)* | | *t*_sex_ (*p*) | | *t*_group_ (*p*) | | *F*_sex-x-group_ (*p*) | |
| Age (years) | | 14.14 (2.17) | 13.96 (2.65) | | 12.79 (2.36) | | 13.36 (2.49) | | **2.96 (.003)** | | -1.63 (.11) | | **2.83 (.025)** | |
| Resilience Score | | -0.04 (0.16) | 0.00 (0.10) | | -0.04 (0.17) | | 0.04 (0.07) | | -1.03 (.31) | | **-2.76 (.007)** | | **2.98 (.020)** | |
| Estimated IQ | | 100.05 (14.39) | 105.56 (10.51) | | 97.25 (12.36) | | 105.77 (12.00) | | 1.54 (.12) | | **-4.84 (< .001)** | | **4.89 (.001)** | |
| Current CD Symptoms | | 3.86 (2.32) | 0.06 (0.27) | | 4.42 (2.13) | | 0.07 (0.30) | | **-4.38 (< .001)** | | **16.59 (< .001)** | | **2.63 (.035)** | |

*Notes.* Sex and group differences were computed using independent samples t-tests. Sex-by-group interactions were computed using univariate analyses of variance. CD, Conduct Disorder; HC, Healthy Controls; IQ, Intelligent Quotient; *M*, Mean; *SD*, Standard Deviation.

|  | |  |  |  |  |  | MNI Coordinates | | | |  |  | |
| --- | --- | --- | --- | --- | --- | --- | --- | --- | --- | --- | --- | --- | --- |
|  | | Brain Region | BA | Hemisphere | NVtxs | Size (mm^2^*)* | x | y | z | | Max | CWP |  |
| *Cortical surface area* | |  |  |  |  |  |  |  |  | |  |  |  |
| Overall positive correlation | | Lateral Occipital Gyrus | 18 | R | 1155 | 932.19 | 29 | -94 | | 11 | 2.41 | 0.004 |  |
|  | |  |  |  |  |  |  |  | |  |  |  |  |
| Overall negative correlation | Inferior Temporal Gyrus | 20 | L | 717 | 734.74 | -30 | -3 | | -44 | -4.31 | 0.027 |  |  |
|  | |  |  |  |  |  |  |  | |  |  |  |  |
| *Local gyrification index* | |  |  |  |  |  |  |  | |  |  |  |  |
| Overall positive correlation | | Superior Frontal Gyrus | 6 | R | 1428 | 853.13 | 17 | 5 | | 65 | 3.17 | 0.003 |  |
|  | |  |  |  |  |  |  |  | |  |  |  |  |
| Females positive,  males negative | | Rostral Anterior Cingulate Cortex | 32 | L | 1473 | 1174.37 | -10 | 42 | | 9 | -2.22 | <0.001 |  |
|  | | Middle Temporal Gyrus | 21 | L | 1050 | 820.77 | -55 | -12 | | -18 | -3.95 | 0.003 |  |
|  | | Medial Orbitofrontal Cortex | 11 | R | 698 | 789.27 | 5 | 58 | | -21 | -2.21 | 0.008 |  |

**Table S3**

*Correlations Between Resilience Scores and Cortical Structure and Sex-by-Resilience Score Interactions when Controlling for IQ (n=284)*

*Notes.* Two participants were excluded from this sensitivity analysis due to missing IQ data. BA, Brodmann Area; CWP, Cluster-Wise P Value; L, Left; Max, Maximum -log10 (p value) in the Cluster; MNI, Montreal Neurological Institute; NVtxs, Number of Vertices; R, Right.

**Table S4**

*Correlations Between Resilience Scores and Sex-by-Resilience Score Interactions in Cortical Structure in the Healthy Controls Only (n=211)*

|  |  |  |  |  |  | MNI Coordinates | | |  |  |
| --- | --- | --- | --- | --- | --- | --- | --- | --- | --- | --- |
|  | Brain Region | BA | Hemisphere | NVtxs | Size (mm^2^*)* | x | y | z | Max | CWP |
| *Cortical surface area* |  |  |  |  |  |  |  |  |  |  |
| Overall negative correlation | Superior Frontal Gyrus | 6 | L | 765 | 727.66 | -23 | 20 | 59 | -2.40 | 0.029 |
|  |  |  |  |  |  |  |  |  |  |  |
| Females positive, males negative | Fusiform Gyrus | 19 | L | 2166 | 1963.90 | -28 | -69 | -16 | -4.48 | <0.001 |
|  | Middle Temporal Gyrus | 21 | L | 878 | 690.89 | -63 | -25 | -10 | -3.55 | 0.041 |
|  |  |  |  |  |  |  |  |  |  |  |
| *Local gyrification index* |  |  |  |  |  |  |  |  |  |  |
| Overall negative correlation | Superior Frontal Gyrus | 9 | R | 1036 | 839.86 | 4 | 45 | 46 | -2.30 | 0.004 |
|  |  |  |  |  |  |  |  |  |  |  |
| Females negative, males positive | Superior Parietal Lobule | 7 | L | 982 | 591.84 | -24 | -62 | 65 | 2.28 | 0.048 |
|  |  |  |  |  |  |  |  |  |  |  |
| Females positive, males negative | Rostral Middle Frontal Gyrus | 10 | L | 1071 | 1231.15 | -29 | 61 | -12 | -3.52 | <0.001 |
|  | Supramarginal Gyrus | 40 | L | 901 | 631.05 | -62 | -33 | 21 | -2.07 | 0.031 |
|  | Inferior Temporal Gyrus | 20 | R | 1777 | 1929.16 | 46 | -2 | -42 | -3.14 | <0.001 |
|  | Superior Frontal Gyrus | 9 | R | 1115 | 960.63 | 4 | 46 | 47 | -2.98 | 0.001 |
|  | Isthmus Cingulate Gyrus | 30 | R | 1511 | 775.74 | 2 | -47 | 19 | -2.92 | 0.009 |
|  | Superior Frontal Gyrus | 6 | R | 909 | 640.92 | 23 | 5 | 67 | -2.59 | 0.030 |
|  |  |  |  |  |  |  |  |  |  |  |
| *Cortical thickness* |  |  |  |  |  |  |  |  |  |  |
| Females positive, males negative | Inferior Temporal Gyrus | 19 | L | 146 | 118.93 | -50 | -67 | -5 | -5.20 | 0.024 |
|  | Lingual Gyrus | 19 | R | 238 | 267.83 | 12 | -66 | -8 | -4.53 | 0.025 |

*Notes.* Results are presented *without* controlling for IQ. BA, Brodmann Area; CWP, Cluster-Wise P Value; L, Left; Max, Maximum -log10 (p value) in the Cluster; MNI, Montreal Neurological Institute; NVtxs, Number of Vertices; R, Right.

**Table S5**

*Correlations Between Resilience Scores and Sex-by-Resilience Score Interactions in Cortical Structure in Participants with CD Only (n=75)*

|  |  |  |  |  |  | MNI Coordinates | | |  |  |
| --- | --- | --- | --- | --- | --- | --- | --- | --- | --- | --- |
|  | Brain Region | BA | Hemisphere | NVtxs | Size (mm^2^*)* | x | y | z | Max | CWP |
| *Local gyrification index* |  |  |  |  |  |  |  |  |  |  |
| Overall negative correlation | Postcentral Gyrus | 40 | R | 2494 | 1307.86 | 62 | -21 | 17 | -2.44 | <0.001 |
|  | Inferior Temporal Gyrus | 20 | R | 1156 | 808.21 | 52 | -41 | -26 | -2.63 | 0.006 |
|  |  |  |  |  |  |  |  |  |  |  |
| Females positive, males negative | Medial Orbitofrontal Cortex | 32 | L | 1562 | 1482.59 | -12 | 45 | -4 | -2.72 | <0.001 |
|  |  |  |  |  |  |  |  |  |  |  |
| *Cortical thickness* |  |  |  |  |  |  |  |  |  |  |
| Females negative, males positive | Superior Temporal Gyrus | 38 | R | 464 | 491.16 | 44 | 21 | -28 | 3.16 | 0.020 |
|  | Superior Frontal Gyrus | 9 | R | 474 | 471.24 | 19 | 46 | 39 | 3.14 | 0.028 |
|  | Lateral Occipital Cortex | 18 | R | 455 | 440.63 | 12 | -98 | 15 | 3.53 | 0.045 |

*Notes.* Results are presented *without* controlling for IQ. BA, Brodmann Area; CD, Conduct Disorder; CWP, Cluster-Wise P Value; L, Left; Max, Maximum -log10 (p value) in the Cluster; MNI, Montreal Neurological Institute; NVtxs, Number of Vertices; R, Right.

**Table S6**

*Correlations Between Resilience Scores and Sex-by-Resilience Score Interactions in Cortical Structure in Participants Classified as either Mid, Late, or Post-Pubertal on the Pubertal Development Scale (n=218)*

|  |  |  |  |  |  | MNI Coordinates | | |  |  |
| --- | --- | --- | --- | --- | --- | --- | --- | --- | --- | --- |
|  | Brain Region | BA | Hemisphere | NVtxs | Size (mm^2^*)* | x | y | z | Max | CWP |
| *Cortical surface area* |  |  |  |  |  |  |  |  |  |  |
| Overall positive correlation | Lateral Occipital Cortex | 18 | R | 1266 | 933.39 | 28 | -88 | 7 | 3.23 | 0.004 |
|  |  |  |  |  |  |  |  |  |  |  |
| Overall negative correlation | Entorhinal Cortex | 38 | L | 631 | 696.21 | -23 | 1 | -42 | -3.46 | 0.039 |
|  |  |  |  |  |  |  |  |  |  |  |
| *Cortical volume* |  |  |  |  |  |  |  |  |  |  |
| Overall positive correlation | Pericalcarine Cortex | 17 | L | 509 | 512.19 | -6 | -75 | 10 | 2.28 | 0.046 |
|  |  |  |  |  |  |  |  |  |  |  |
| *Local gyrification index* |  |  |  |  |  |  |  |  |  |  |
| Overall positive correlation | Lateral Orbitofrontal Cortex | 47 | R | 917 | 709.14 | 44 | 28 | -17 | 3.04 | 0.014 |
|  |  |  |  |  |  |  |  |  |  |  |
| Females positive,  males negative | Rostral Anterior Cingulate Cortex | 32 | L | 2528 | 2009.10 | -9 | 43 | 7 | -3.08 | <0.001 |
|  | Middle Temporal Gyrus | 21 | L | 1540 | 1241.88 | -56 | -13 | -18 | -5.11 | <0.001 |
|  | Medial Orbitofrontal Cortex | 10 | R | 667 | 730.84 | 5 | 59 | -7 | -2.12 | 0.012 |

*Notes.* Results are presented *without* controlling for IQ. BA, Brodmann Area; CWP, Cluster-Wise P Value; L, Left; Max, Maximum -log10 (p value) in the Cluster; MNI, Montreal Neurological Institute; NVtxs, Number of Vertices; R, Right.
